# Analytical model of reactive transport processes with spatially variable coefficients

**DOI:** 10.1098/rsos.140348

**Published:** 2015-05-20

**Authors:** Matthew J. Simpson, Liam C. Morrow

**Affiliations:** School of Mathematics, Queensland University of Technology, Brisbane, Queensland, Australia

**Keywords:** contaminant transport, saturated porous media, analytical model, partial differential equation, symbolic computation

## Abstract

Analytical solutions of partial differential equation (PDE) models describing reactive transport phenomena in saturated porous media are often used as screening tools to provide insight into contaminant fate and transport processes. While many practical modelling scenarios involve spatially variable coefficients, such as spatially variable flow velocity, *v*(*x*), or spatially variable decay rate, *k*(*x*), most analytical models deal with constant coefficients. Here we present a framework for constructing exact solutions of PDE models of reactive transport. Our approach is relevant for advection-dominant problems, and is based on a regular perturbation technique. We present a description of the solution technique for a range of one-dimensional scenarios involving constant and variable coefficients, and we show that the solutions compare well with numerical approximations. Our general approach applies to a range of initial conditions and various forms of *v*(*x*) and *k*(*x*). Instead of simply documenting specific solutions for particular cases, we present a symbolic worksheet, as supplementary material, which enables the solution to be evaluated for different choices of the initial condition, *v*(*x*) and *k*(*x*). We also discuss how the technique generalizes to apply to models of coupled multispecies reactive transport as well as higher dimensional problems.

## 2. Introduction

Exact analytical solutions of partial differential equation (PDE) models describing reactive transport phenomena in saturated porous media are of interest for several reasons. First, exact solutions are commonly used as screening tools to provide preliminary insight into management scenarios [1–3]. Second, unlike numerical solutions, exact solutions are often simple to evaluate computationally which is particularly important when implementing an inverse technique for model calibration [4]. Finally, exact solutions are of particular interest under advection-dominant conditions where standard numerical methods can suffer from artificial oscillations [5].

While the literature contains a large number of exact solutions of reactive transport PDEs, e.g. [6–13], many of these solutions are limited to relatively simple scenarios involving constant transport and reaction rates, one-dimensional flow conditions, single species reactive transport or relatively simple (solute-free) initial conditions. While some exact solutions have considered spatially variable transport terms, these previous studies have not considered spatially variable reaction rates [11] or coupled multispecies reaction networks [14,15].

In this brief report, we present a framework for constructing analytical solutions of a general class of reactive transport PDE models. Based on a perturbation technique [16,17] and the method of characteristics [18], the framework is relevant for advection-dominant conditions, which is of interest as standard numerical solutions can be limited in such situations [5]. Our approach uses regular perturbation theory, which relies on the fact that the governing equations contain a small parameter and that we can obtain an exact solution to the governing equations when the small parameter is set to zero. We then construct an approximate solution to the governing equations as a power series in the small parameter [16,17].

Our approach is sufficiently general that it applies to reactive transport PDEs with (i) spatially variable coefficients, (ii) coupled multispecies reaction networks, (iii) multi-dimensional flow, and (iv) practical initial conditions where traditional integral transform techniques do not apply [9]. Here, we present the key features of the technique for a range of one-dimensional single species reactive transport PDE models, including both constant and spatially variable coefficients. We also describe how the approach applies to several extensions including coupled multispecies reactive transport models and multi-dimensional reactive transport models. To keep this brief report as concise as possible, all symbolic algorithms used to construct these solutions are presented as electronic supplementary material.

## 3. Methods

We will construct a solution of

3.1 $$\frac{\partial C}{\partial t}=\frac{\partial }{\partial x}\left[D(x)\frac{\partial C}{\partial x}\right]-\frac{\partial }{\partial x}[v(x)C]-k(x)C,$$

on −∞<x<∞, with the initial condition *C*(*x*,0)=*g*(*x*) and boundary conditions $\lim_{x\to \pm \infty }C(x,t)=0$. Here, *D*(*x*)[*L*^2^*T*^−1^] is the dispersion coefficient, *v*(*x*)[*LT*^−1^] is the advective velocity and *k*(*x*)[*T*^−1^] is the decay coefficient. We seek such a solution under the condition that the dispersion term is small and we have D(x)≡D=O(ε). The perturbation solution is of the form $C(x,t)=\sum_{j=0}^{\infty }C_{j}(x,t)\varepsilon^{j}$ [16,17], and we will discuss the implications of making such an assumption in the Results section. Substituting $C(x,t)=\sum_{j=0}^{\infty }C_{j}(x,t)\varepsilon^{j}$ into equation (3.1) and equating powers of *ε* gives us

3.2 $$\frac{\partial C_{0}}{\partial t}=-\frac{\partial }{\partial x}[v(x)C_{0}]-k(x)C_{0},\;O(1)$$

and

3.3 $$\frac{\partial C_{j}}{\partial t}=\frac{\partial }{\partial x}\left[D(x)\frac{\partial C_{j-1}}{\partial x}\right]-\frac{\partial }{\partial x}[v(x)C_{j}]-k(x)C_{j},\;O(\varepsilon^{j}),\;\text{for}\;j=1,2,3,\ldots .$$

To match the initial condition for equation (3.1), we set *C*_0_(*x*,0)=*g*(*x*) and *C*_*j*_(*x*,0)=0 for *j*=1,2,3,…, and the boundary conditions are $\lim_{x\to \pm \infty }C_{j}(x,t)=0$ for *j*=0,1,2,3,…. This family of PDEs can be solved exactly to give *C*_*j*_(*x*,*t*) for *j*=0,1,2,3,…, using the method of characteristics [18,19]. We now describe the details of this strategy for four particular cases.

### 3.1 Case 1: constant coefficients

First, we solve equation (3.1) with constant coefficients *D*(*x*)=*D*, *v*(*x*)=*v* and *k*(*x*)=*k*. Under these conditions the solution of the O(1) equation is given by the method of characteristics. Along the characteristic curves given by d*x*/d*t*=*v*, the PDE governing *C*_0_(*x*,*t*) simplifies to d*C*_0_/d*t*=−*kC*_0_. The solution of these two ordinary differential equations (ODEs) allows us to write *C*_0_(*x*,*t*)=*g*(*x*−*vt*) exp(−*kt*). We also solve the $O(\varepsilon^{j})$ equations using the method of characteristics by recognizing that when we set d*x*/d*t*=*v*, the PDE governing *C*_*j*_(*x*,*t*) simplifies to an ODE, d*C*_*j*_/d*t*=−*kC*_*j*_+∂^2^*C*_*j*−1_/∂*x*^2^, which can be solved using an integrating factor. The solutions can be written as

3.4 $$\left.\begin{array}{rl} C_{0}(x,t) & =g(x-vt)\;\text{exp}(-kt)\\ \;\text{and}\;C_{j}(x,t) & =\text{exp}(-kt)\int \text{exp}(kt)\frac{\partial^{2}C_{j-1}(x-vt,t)}{\partial x^{2}}\;\text{d}t,\;\text{for}\;j=1,2,3,\ldots . \end{array}\right\}$$

Expressions for *C*_*j*_(*x*,*t*) depend on the initial condition, *g*(*x*). For many practical problems we are often interested in an initial condition representing a localized plume of dissolved solute within a larger, solute-free region [20–22]. To mimic this, we choose the initial condition to be Gaussian, *g*(*x*)=exp(−[(*x*−*x*_0_)/*l*]^2^), corresponding to a plume of width *l*, centred at *x*=*x*_0_. For this initial condition, the solution is given by

3.5 $$\begin{array}{rl} C(x,t) & =\text{exp}\left(\frac{\xi^{2}}{l^{2}}-kt\right)-\frac{2\varepsilon }{l^{4}}\;\text{exp}\left(\frac{\xi^{2}}{l^{2}}-kt\right)t(l^{2}-2\xi^{2})\\ & \;+\frac{2\varepsilon^{2}}{l^{8}}\;\text{exp}\left(\frac{\xi^{2}}{l^{2}}-kt\right)t^{2}(3l^{4}+12\xi^{2}l^{2}-4\xi^{4})+O(\varepsilon^{3}), \end{array}$$

where *ξ*=*x*−*x*_0_−*vt*. It is possible to find additional terms in the perturbation series, or to use this technique to solve equation (3.1) for different *g*(*x*). Instead of listing further terms in the perturbation solution, or further solutions for different *g*(*x*), we present a general Maple symbolic worksheet which automates this process (electronic supplementary material).

### 3.2 Case 2: spatially variable decay rate

We now demonstrate how to find exact solutions of equation (3.1) for problems with a spatially variable reaction rate, *k*(*x*), but we maintain constant dispersion and advective velocity, *D*(*x*)=*D* and *v*(*x*)=*v*. The solution of the O(1) equation is given by recognizing that when d*x*/d*t*=*v* the PDE governing *C*_0_(*x*,*t*) simplifies to an ODE, d*C*_0_/d*t*=−*k*(*x*)*C*_0_. The solution of these two ODEs gives *C*_0_(*x*,*t*)=*g*(*x*−*vt*) exp(−*k*(*x*)*t*). To solve the O(ε) equation, we again apply the method of characteristics. Setting d*x*/d*t*=*v*, the PDE governing *C*_1_(*x*,*t*) simplifies to d*C*_1_/d*t*=−*kC*_1_+∂^2^*C*_0_/∂*x*^2^, which can be solved using an integrating factor. The solutions of these two ODEs can be combined to give *C*_1_(*x*,*t*). Although it is possible to solve these differential equations for particular choices of *g*(*x*) and *k*(*x*), and to report a truncated perturbation solution for *C*(*x*,*t*), we prefer to keep this report as brief as possible and present a general Maple worksheet which automates the evaluation of such solutions for different choices of *k*(*x*) and *g*(*x*).

### 3.3 Case 3: spatially variable advection velocity

We also demonstrate how find exact solutions of equation (3.1) for problems where we have spatially variable advection rate, *v*(*x*), but we maintain constant dispersion and reaction rate, *D*(*x*)=*D* and *k*(*x*)=*k*. The solution of the O(1) equation is given by recognizing that when d*x*/d*t*=*v*(*x*) the PDE governing *C*_0_(*x*,*t*) simplifies to an ODE, d*C*_0_/d*t*=−*C*_0_(*k*+d*v*/d*x*). For a particular choice of *v*(*x*), these ODEs can be solved and the two solutions used to give *C*_0_(*x*,*t*). Similarly, the equations governing *C*_1_(*x*,*t*) can be solved using the method of characteristics and we present a general Maple worksheet which automates the evaluation of such exact solutions for different choices of *v*(*x*) and *g*(*x*).

### 3.4 Case 4: coupled multispecies reactive transport

In some situations, it is relevant to implement an extension of equation (3.1) by considering the transport and reaction of multiple species coupled through some kind of reaction network [7,8,23,24]. Our solution strategy is also relevant for these extensions and to demonstrate the key features of how our approach applies we will consider a two species model, which can be written as

3.6 $$\left.\begin{array}{rl} R\frac{\partial C^{1}}{\partial t} & =\frac{\partial }{\partial x}\left[D(x)\frac{\partial C^{1}}{\partial x}\right]-\frac{\partial }{\partial x}[v(x)C^{1}]-k^{1}(x)C^{1}\\ \;\text{and}\;\frac{\partial C^{2}}{\partial t} & =\frac{\partial }{\partial x}\left[D(x)\frac{\partial C^{2}}{\partial x}\right]-\frac{\partial }{\partial x}[v(x)C^{2}]+k^{1}(x)C^{1}-k^{2}(x)C^{2}, \end{array}\right\}$$

where *C*^1^(*x*,*t*) is the concentration of the parent species and *C*^2^(*x*,*t*) is the concentration of the daughter species. The retardation factor, *R*≥1 [−], represents a linear equilibrium reaction [4], *k*^1^(*x*) [*T*^−1^] is the reaction rate describing the decay of the parent species into the daughter species and *k*^2^(*x*) [*T*^−1^] is the reaction rate describing the decay of the daughter species. We consider an initial condition *C*^1^(*x*,0)=*g*^1^(*x*) and *C*^2^(*x*,0)=*g*^2^(*x*). Our approach for solving the coupled problem follows directly from the single species model as we seek a solution of the form $C^{1}(x,t)=\sum_{j=0}^{\infty }C_{j}^{1}(x,t)\varepsilon^{j}$ and $C^{2}(x,t)=\sum_{j=0}^{\infty }C_{j}^{2}(x,t)\varepsilon^{j}$, with D(x)≡D=O(ε). Substituting these series into equation (3.6) and equating coefficients of *ε* gives a family of hyperbolic PDEs which can be solved using the method of characteristics. To briefly demonstrate the salient features of the solution strategy, we consider a case with constant coefficients *D*(*x*)=*D*, *v*(*x*)=*v*, *k*^1^(*x*)=*k*^1^ and *k*^2^(*x*)=*k*^2^. The O(1) governing equations are given by

3.7 $$\left.\begin{array}{rl} R\frac{\partial C_{0}^{1}}{\partial t} & =-v\frac{\partial C_{0}^{1}}{\partial x}-k^{1}C_{0}^{1}\\ \;\text{and}\;\frac{\partial C_{0}^{2}}{\partial t} & =-v\frac{\partial C_{0}^{2}}{\partial x}+k^{1}C_{0}^{1}-k^{2}C_{0}^{2}, \end{array}\right\}$$

with $C_{0}^{1}(x,0)=g^{1}(x)$ and $C_{0}^{2}(x,0)=g^{2}(x)$. The solution of these coupled PDEs can be found using the method of characteristics. For example, with *g*^1^(*x*)=exp(−[(*x*−*x*_0_)/*l*]^2^) and *g*^2^(*x*)=0, we have $C_{0}^{1}(x,t)=\text{exp}(-{\left[(x-tv/R-x_{0})/l\right]}^{2}-(k_{1}/R)t)$ and a complicated expression for $C_{0}^{2}(x,t)$ in terms of error functions. Instead of documenting these solutions here, we present a symbolic worksheet (electronic supplementary material) which can be used to calculate $C_{0}^{1}(x,t)$, $C_{0}^{2}(x,t)$, $C_{1}^{1}(x,t)$, $C_{2}^{1}(x,t)$ and so forth. This symbolic worksheet also applies to situations where we have spatially variable transport and reaction rates.

## 4. Results

To confirm the accuracy of the proposed method, we compare the perturbation solutions with numerical solutions of equation (3.1) and equation (3.6). The numerical solutions are obtained using a finite difference approximation, with central differences, on a uniformly discretized mesh with spacing *δx*. We integrate the resulting system of coupled ordinary differential equations using implicit Euler time stepping with time steps of duration *δt*. At each time step, the resulting system of linear equations is solved using the Thomas algorithm [4,5].

**Figure 1.**
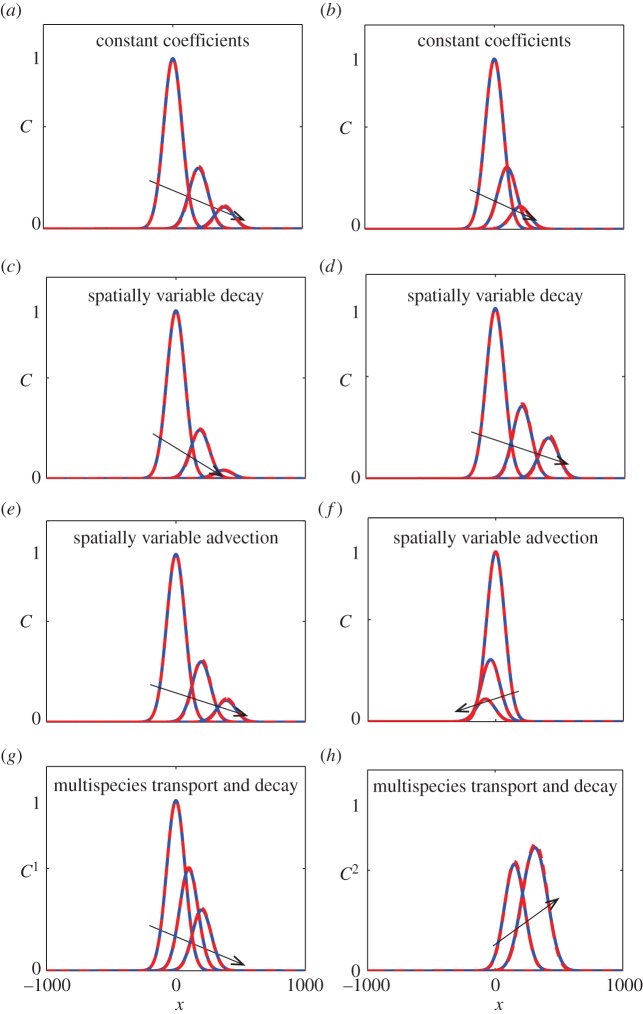
(*a*–*f*) Comparison of the O(ε) perturbation solution (red dashed) and a numerical solution (blue solid) of equation (3.1) with *C*(*x*,0)= exp(−[(*x*−*x*_0_)/*l*]^2^), with *x*_0_=0 and *l*=100. The comparison in (*a*,*b*) corresponds to equation (3.1) with constant coefficients with *D*=1, *k*=0.05, with (*a*) *v*=10 and (*b*) *v*=5. The comparison in (*c*,*d*) corresponds to equation (3.1) with a spatially variable decay date, *D*=1 and *v*=10. Results are presented for *k*(*x*)= *α* exp(*βx*), with (*c*) *α*=0.05 and *β*=0.002, and (*d*) *α*=0.05 and *β*=−0.002. The comparison in (*e*,*f*) corresponds to equation (3.1) with a spatially variable advection velocity, *D*=1 and *k*=0.05. Results are presented for *v*(*x*)=*α*/(*β*+*x*), with (*c*) *α*=100 000 and *β*=10 000, and (*c*) *α*=−100 000 and *β*=50 000. (*g*,*h*) Comparison of the O(ε) perturbation solution (red dashed) and a numerical solution (blue solid) of equation (3.6) with *C*^1^(*x*,0)= exp(−[(*x*−*x*_0_)/*l*]^2^), and *C*^2^(*x*,0)=0 and *x*_0_=0 and *l*=100. For the coupled problem, we have *R*=2, *v*=10, *D*=1, *k*_1_=0.05 and *k*_2_=0.01. All subfigures show solutions at *t*=0, 20 and 40, with the direction of increasing *t* denoted by the arrows. Numerical solutions correspond to *δx*=0.1 and *δt*=0.1, on the truncated domain −1000≤*x*≤1000 with homogeneous Dirichlet boundary conditions.

Results in figure 1*a*,*b* compare perturbation and numerical solutions of equation (3.1) for the case where we have constant coefficients, indicating that the perturbation solution is visually indistinguishable from the numerical solution at this scale. The two sets of results differ only the rate of advection, *v*, and in both cases we clearly see the impact of both the transport and decay of the initial plume as it spreads and decays with time. Results in figure 1*c*,*d* compare perturbation and numerical solutions of equation (3.1) where we have a spatially variable decay rate, *k*(*x*). The profiles in figure 1*c* correspond to an increasing rate of decay, d*k*(*x*)/d*x*>0, whereas the profiles in figure 1*d* correspond to a decreasing rate of decay, d*k*(*x*)/d*x*<0. The impact of these differences is very clear when comparing the results in figure 1*c*,*d* as the effect of decay is more pronounced in figure 1*c* where *k* increases with *x*. These kinds of situations, where the rate of decay varies with position, have been studied previously using numerical models [25] but are not routinely incorporated into analytical models. Results in figure 1*e*,*f* compare perturbation and numerical solutions of equation (3.1) where we have a spatially variable advection velocity, *v*(*x*), which is relevant to situations involving diverging or converging flow, such as in the vicinity of a pumping well [11]. The profiles in figure 1*e* correspond to a spatially variable advection velocity with *v*(*x*)>0, whereas the profiles in figure 1*f* correspond to a spatially variable advection velocity with *v*(*x*)<0. The impact of these differences is very clear since the initial plume in figure 1*e* is transported in the positive *x* direction, whereas the initial plume in figure 1*f* is transported in the negative *x* direction. Results in figure 1*g*,*h* compare perturbation and numerical solutions of equation (3.6). The profiles in figure 1*g* correspond to the parent species, *C*^1^(*x*,*t*), which is simultaneously transported while decaying into the daughter species, *C*^2^(*x*,*t*), which is shown in figure 1*h*. In all cases, the perturbation solution compares very well with the numerical solutions.

All results presented in figure 1 correspond to a particular initial condition and several particular choices of *v*(*x*) and *k*(*x*). While our solution strategy is relevant for different choices of the initial condition, *v*(*x*) and *k*(*x*), instead of simply documenting these additional results here, we chose to present a symbolic computational worksheet (electronic supplementary material) which can be used to generate further solutions. While the perturbation solutions in figure 1 correspond to an O(ε) approximation, it is possible to calculate higher order perturbation solutions. For example, the Maple worksheets (electronic supplementary material) can be used to calculate the next, $O(\varepsilon^{2})$ term in the perturbation series and all worksheets can be further modified to calculate additional terms, if required.

## 5. Discussion and conclusion

Analytical solutions of PDE models describing reactive transport processes have several practical uses, such as screening tools to provide preliminary insight into contaminant fate and transport [1,2]. While the literature contains a large number of exact solutions, many of these are limited to relatively simple scenarios involving constant transport and reaction rates, single species processes or relatively simple (solute-free) initial conditions. In this work, we have sought to develop, describe and test a framework for constructing exact solutions of reactive transport PDEs which can incorporate spatially variable coefficients, multispecies decay chain processes and more practical initial conditions than is often possible using traditional integral transform techniques. The solutions developed here are relevant for advection-dominant conditions since we make the simplifying assumption that the dispersion term in the governing equations is small, allowing the impact of dispersion to be incorporated into the solution through a regular perturbation problem by solving a family of first order PDEs using the method of characteristics. Unlike other methods based on an integral transform technique, our approach can be implemented for a wide range of initial conditions. Instead of documenting solutions relevant for one or two initial conditions only, we prefer to implement the solution strategy here in a symbolic framework so that others can implement the solution strategy for varying conditions. For example, the symbolic worksheets (electronic supplementary material) can be used to construct perturbation solutions for varying initial conditions and different functional forms of *v*(*x*) and *k*(*x*). The symbolic worksheets contain sufficient information to construct the O(1) and O(ε) terms in the perturbation series, and it is straightforward to make further adjustments to the worksheets such that higher order correction terms can also be calculated, if required. There are several practical examples where spatially variable coefficients in the governing reactive transport equation are relevant such as converging or diverging flow near a pumping well [11,26], or reactive transport in complicated geological settings where the decay rate is thought to vary spatially because of the underlying geological or geochemical conditions [25].

The accuracy of the perturbation solutions presented in this brief report was examined by comparing them with numerical solutions. In all cases, we compared an O(ε) perturbation solution with a numerical solution and found that, with *D*=1, the two solutions compared well, as shown in figure 1. We also compared perturbation and numerical solutions for other parameter choices, and in particular, we explored how large *D* could be made before the O(ε) perturbation solution no longer matched the numerical solutions. Repeating the comparisons in figure 1 with *D*=5 gave a good match between the perturbation and numerical solutions (not shown), whereas with *D*=10 the O(ε) perturbation solution did not match (not shown). Of course, the accuracy of the perturbation solution can be improved by retaining further terms in the series, if required.

While the main part of this brief report has focused on solving one-dimensional reactive transport problems, our approach is also relevant for higher dimensional problems; for example, we also present (electronic supplementary material) a symbolic worksheet that can be used to construct such solutions for a single species, constant coefficient, two-dimensional reactive transport PDE. Further adaptations of the perturbation solution method for higher-dimensional reactive transport PDEs, such as including spatially variable coefficients or generalization to multispecies problems, follow directly from the one-dimensional results. In addition, all results presented here correspond to the case where the dispersion term is constant and we have *D*(*x*)=*D*, with D=O(ε). This assumption can also be relaxed by considering D(x)=D(x)D, with D=O(ε), and specifying some spatial distribution given by D(x). While we have focused on solving problems on an infinite domain, −∞<x<∞, effectively obviating the explicit need for incorporating boundary conditions, the solution strategy we outline here can also be used on a semi-infinite or finite domain by using standard techniques to incorporate boundary condition information into the method of characteristics [18].

## Supplementary Material

File 1: Maple worksheet to construct exact solutions for one-dimensional, single species, reactive transport models.

## Supplementary Material

File 2: Maple worksheet to construct exact solutions for one-dimensional, coupled multispecies, reactive transport models.

## Supplementary Material

File 3: Maple worksheet to construct exact solutions for two-dimensional, single species, reactive transport models.

## Data accessibility

All experimental data required to repeat the analysis in this article are included within the article.
